# Fine structure of synapses on dendritic spines

**DOI:** 10.3389/fnana.2014.00094

**Published:** 2014-09-09

**Authors:** Michael Frotscher, Daniel Studer, Werner Graber, Xuejun Chai, Sigrun Nestel, Shanting Zhao

**Affiliations:** ^1^Institute for Structural Neurobiology, Center for Molecular Neurobiology Hamburg, University Medical Center Hamburg-EppendorfHamburg Germany; ^2^Institute of Anatomy, University of BernBern Switzerland; ^3^Institute of Anatomy and Cell Biology, University of FreiburgFreiburg Germany

**Keywords:** dendritic spine, cofilin, actin cytoskeleton, immunogold labeling, electron microscopy, high-pressure freezing

## Abstract

Camillo Golgi’s “Reazione Nera” led to the discovery of dendritic spines, small appendages originating from dendritic shafts. With the advent of electron microscopy (EM) they were identified as sites of synaptic contact. Later it was found that changes in synaptic strength were associated with changes in the shape of dendritic spines. While live-cell imaging was advantageous in monitoring the time course of such changes in spine structure, EM is still the best method for the simultaneous visualization of all cellular components, including actual synaptic contacts, at high resolution. Immunogold labeling for EM reveals the precise localization of molecules in relation to synaptic structures. Previous EM studies of spines and synapses were performed in tissue subjected to aldehyde fixation and dehydration in ethanol, which is associated with protein denaturation and tissue shrinkage. It has remained an issue to what extent fine structural details are preserved when subjecting the tissue to these procedures. In the present review, we report recent studies on the fine structure of spines and synapses using high-pressure freezing (HPF), which avoids protein denaturation by aldehydes and results in an excellent preservation of ultrastructural detail. In these studies, HPF was used to monitor subtle fine-structural changes in spine shape associated with chemically induced long-term potentiation (cLTP) at identified hippocampal mossy fiber synapses. Changes in spine shape result from reorganization of the actin cytoskeleton. We report that cLTP was associated with decreased immunogold labeling for phosphorylated cofilin (p-cofilin), an actin-depolymerizing protein. Phosphorylation of cofilin renders it unable to depolymerize F-actin, which stabilizes the actin cytoskeleton. Decreased levels of p-cofilin, in turn, suggest increased actin turnover, possibly underlying the changes in spine shape associated with cLTP. The findings reviewed here establish HPF as an appropriate method for studying the fine structure and molecular composition of synapses on dendritic spines.

## INTRODUCTION

Early studies by Ramón y Cajal (1911) and his contemporaries already indicated that dendritic spines are not artifacts of the silver impregnation technique introduced by Camillo Golgi (Golgi, 1873). Querton (1898) observed changes in dendritic spines during hibernation, suggesting that dendritic spines are plastic structural elements that are subject to modification in an activity-dependent manner. By means of electron microscopy (EM) Gray (1959) showed that spines are postsynaptic elements, indicating that changes in spine number reflect changes in the connectivity pattern of the neuronal network. While studying network plasticity, many authors reported that afferent denervation affected the number of dendritic spines on postsynaptic cells. Thus, enucleation or dark rearing was found to result in a loss of dendritic spines in Golgi-impregnated neurons of the visual cortex (e.g., Valverde, 1967, 1968; Fifková, 1970). In contrast, the stimulation of an animal increased the number of dendritic spines and synapses (see Shapiro and Vukovic, 1970; Frotscher et al., 1975; Greenough et al., 1985). As far as the complex spines postsynaptic to hippocampal mossy fiber boutons (MFB) are concerned, we showed some time ago that removal of the entorhinal cortex during development results in *trans*-synaptic malformation of these spines (Frotscher et al., 1977). Collectively, these findings pointed to an inductive role of afferent fibers in the formation of their postsynaptic elements (Hámori, 1973). These observations in fixed tissue were supported by real-time microscopy studies showing *de novo* formation of dendritic spines following long-term potentiation (LTP) induction (Engert and Bonhoeffer, 1999). Recent studies further revealed that the structure of individual spines is not static but subject to change. Thin, long spines were assumed to be nascent spines, compared to the large mushroom-shaped spines that were associated with memory traces (Matsuzaki et al., 2004). Thus, the different spine categories, thin, stubby, and mushroom-shaped (Peters and Kaiserman-Abramof, 1970), appeared to represent different functional states. Theoretical considerations, as well as experimental data, led to the conclusion that dendritic spines are small devices that subserve chemical compartmentalization by amplifying and isolating synaptically induced second messengers such as calcium (Koch et al., 1992).

Changes in spine structure were accompanied by changes in the actin cytoskeleton and shifts in the equilibrium between F-actin and G-actin (Okamoto et al., 2004) by virtue of actin-depolymerizing proteins such as cofilin. The severing activity of cofilin is terminated by phosphorylation of the protein (Arber et al., 1998; Yang et al., 1998), and increased spine size in LTP was found to be associated with increased phosphorylation of cofilin (Chen et al., 2007; Rex et al., 2009), indicating stabilization of the spine cytoskeleton.

Several groups have reported on the changes in synaptic ultrastructure following LTP induction (Desmond and Levy, 1988; Buchs and Muller, 1996; Toni et al., 1999; Geinisman, 2000; Harris et al., 2003). Collectively, these studies indicated restructuring of synapses following LTP induction, ranging from synapse enlargement to the formation of spine-like protrusions.

With the present review article we pursue the following aims: first, we briefly summarize the literature on the fine structure of synapses on dendritic spines. Naturally, such a survey can only highlight some subjectively selected papers and remains incomplete. Second, we address the issue of tissue preservation for EM. We summarize recent studies in which high-pressure freezing (HPF) was used as an alternative method to conventional aldehyde fixation of neural tissue. Third, we report on the use of HPF in EM immunogold labeling studies. Without fixation in aldehyde solution, antigenicity to phosphorylated cofilin (p-cofilin) was found to be much better preserved than after conventional fixation for EM (Studer et al., 2014). Thus, this review summarizes our knowledge on spine synapses as well summarizing recent attempts to improve the preservation of their fine structure and molecular composition.

## FINE STRUCTURE OF SYNAPSES ON DENDRITIC SPINES

Palade and Palay (1954) and Palay (1956, 1958) were the first to describe synapses by using electron microscopic methods. However, it was Gray (1959) who clearly showed that the heads of dendritic spines were postsynaptic elements of asymmetric synapses. He called them “asymmetric” because their postsynaptic density was thicker than the presynaptic membrane specialization. He differentiated them from the “symmetric” synapses often found on cell bodies. Today, we know that the presynaptic boutons of asymmetric synapses on spines contain the excitatory neurotransmitter glutamate, whereas the symmetric contacts formed on cell bodies are GABAergic inhibitory synapses. Harris and Weinberg (2012) have recently provided a comprehensive survey of the ultrastructure of synapses in the mammalian brain.

George Gray also discovered the spine apparatus, an enigmatic organelle consisting of sacs of endoplasmic reticulum intervened by electron dense bars (Gray, 1959). A spine apparatus is found in many, but not all, spines of forebrain neurons. It is absent in spines of cerebellar Purkinje cells (Peters et al., 1991). More recent studies have provided evidence that the spine apparatus is involved in synaptic plasticity and learning and memory. Thus, mouse mutants deficient in synaptopodin, a protein present in renal podocytes and dendritic spines (Mundel et al., 1997; Deller et al., 2000), do not form this organelle and are impaired in LTP and spatial learning (Deller et al., 2003).

Spine counts roughly reveal the number of excitatory synapses on dendritic spines, but there is no one-to-one relationship. In addition to the synapse on the spine head, there may be contacts on the spine neck (Peters and Kaiserman-Abramof, 1970), and the presynaptic bouton of this second synapse may contain a different neurotransmitter, for instance acetylcholine (Frotscher and Léránth, 1986).

In addition to their asymmetric membrane specializations and the presynaptic vesicle-filled bouton, synapses on spines are recognizable by a widening of the extracellular space at the site of the contact, i.e., the synaptic cleft. Synaptic contact zones or release sites may be perforated by a small protrusion of the spine, the spinule (Westrum and Blackstad, 1962; Tarrant and Routtenberg, 1977; Sorra et al., 1998), assumed to be involved in *trans*-endocytosis (Spacek and Harris, 2004). As first described by Steward and Levy (1982), ribosomes are often found at the base of spines or near synaptic contacts, suggesting local protein synthesis.

The fine structural characteristics of spine synapses briefly described here were observed in sections of a thickness of about 50 nm. The heads of large dendritic spines have a diameter of up to 1 μm and are thus much larger. This implies that conventional thin sections only show a small fraction of the spine, its synapses and organelles – unless three-dimensional reconstructions from complete series of thin sections were performed. This is relevant for conclusions regarding the regular occurrence of organelles in a spine or the frequency of synaptic contacts, and one should therefore rather talk of spine profiles when looking at single sections. Moreover, with the exception of some structurally unique synapses, in single sections we hardly know what the presynaptic and postsynaptic partners are. We do not know the functional history of a given spine or spine profile, nor do we know its age. Finally, we cannot be sure that all structures we see represent their native state in the living animal, which needs to be anesthetized and fixed with aldehyde solutions for subsequent EM. Given all these obstacles, it is remarkable that thorough EM analyses provided reliable data on structural changes at spine synapses associated with functional synaptic plasticity such as LTP. Intuitively, one would assume that synaptic strengthening is not only associated with a *de novo* formation of dendritic spines (Engert and Bonhoeffer, 1999) but also with an increase in the number of synaptic contacts. Remarkably, most investigators using conventional fixation and embedding procedures found neither an increase in synapse number (Geinisman, 2000) nor splitting of spines (Harris et al., 2003), but modification of preexisting contacts. For instance, Desmond and Levy (1988) reported an increase of synaptic interface surface area in LTP, and Geinisman (2000) showed an increase in the number of perforated synapses at the expense of non-perforated synapses. Harris et al. (2003) provided evidence of the formation of small spine-like protrusions that encountered presynaptic boutons already synapsing with neighboring spines. Toni et al. (1999) used calcium accumulation to determine activated synapses and observed an increase in the proportion of axon terminals contacting two or more dendritic spines.

A couple of questions arise at this point: How can we reliably identify potentiated synapses in a volume of tissue? How can we reliably identify the presynaptic and the postsynaptic neuron of a given synaptic contact? Can we optimize tissue fixation and dehydration to minimize protein denaturation and shrinkage?

## HIGH-PRESSURE FREEZING AS AN ALTERNATIVE TO CONVENTIONAL ALDEHYDE FIXATION FOR EM

In conventional EM the tissue is subjected to chemical fixation using aldehyde solutions and is dehydrated in ascending series of ethanol. These steps are associated with protein denaturation and tissue shrinkage, respectively, depending on the water content of the various tissue components. Little information is available in the literature on how quickly the tissue is fixed upon exposition to the aldehyde solution. In addition, perfusion of an animal with fixation solution requires anesthesia, opening of the chest, and rinsing of the circulatory system with a rinsing solution before the fixative is administered. It is unknown to what extent these manipulations interfere with the preservation of fine-structural detail, in particular with the preservation of subtle structural changes induced by a preceding stimulation experiment. As far as dendritic spines are concerned, it has been previously shown, using real-time microscopy, that they undergo structural changes upon stimulation (Matsuzaki et al., 2004), but we do not know whether subtle stimulation-induced ultrastructural changes in spine shape, in synaptic structure, and in the molecular composition of spine synapses are preserved by conventional fixation and dehydration procedures.

One way to avoid conventional fixation and dehydration is by shock-freezing the tissue under high pressure, followed by cryosubstitution of the tissue water. HPF immobilizes the tissue in less than a second, resulting in tissue vitrification and excellent preservation of ultrastructural detail (Studer et al., 2001, 2014). Following HPF, the samples are collected in liquid nitrogen and then transferred to the freeze-substitution device. There, the tissue water is substituted by acetone and the sample osmicated for embedding in Epon. For EM immunogold labeling, the water is substituted by methanol (without osmication) and the sample embedded in Lowicryl HM20 (for details see Studer et al., 2014).

High-pressure freezing has been developed since rapid freezing without pressure only preserved the very surface of the tissue, whereas more remote portions showed ice crystal formation damaging cells and organelles. Shock-freezing of the tissue surface was pioneered by Van Harreveld et al. (1965), who studied the extracellular space in central nervous tissue. Heuser et al. (1979) dropped the tissue on a cooled metal block to capture synaptic vesicle fusion. Freezing under high pressure, however, lowers the freezing point of water and increases vitrification depth 10-fold (Sartori et al., 1993). Although HPF thus seems to be a clear improvement when compared to chemical fixation using aldehydes, we do not want to conceal the problems associated with this approach. First, tissue samples need to be small and easily accessible to be rapidly frozen. This may turn out to be critical when the sample needs to be transferred from the recording chamber to the high-pressure freezer, which causes a time delay. Moreover, in order to fit into the cavity of the specimen carrier, the region of interest might have to be punched out from a larger tissue sample. Ice crystal formation may occur when the samples or the instruments to handle them during further preparation are not properly cooled. Finally, warming up in an organic solvent prior to polymerization may lead to a reorganization and loss of some proteins (see Studer et al., 2014, for critical steps of the HPF procedure). Taken together, HPF is an alternative to conventional aldehyde fixation, but application of this complex method makes it necessary to carefully select the questions to be addressed.

**FIGURE 1:**
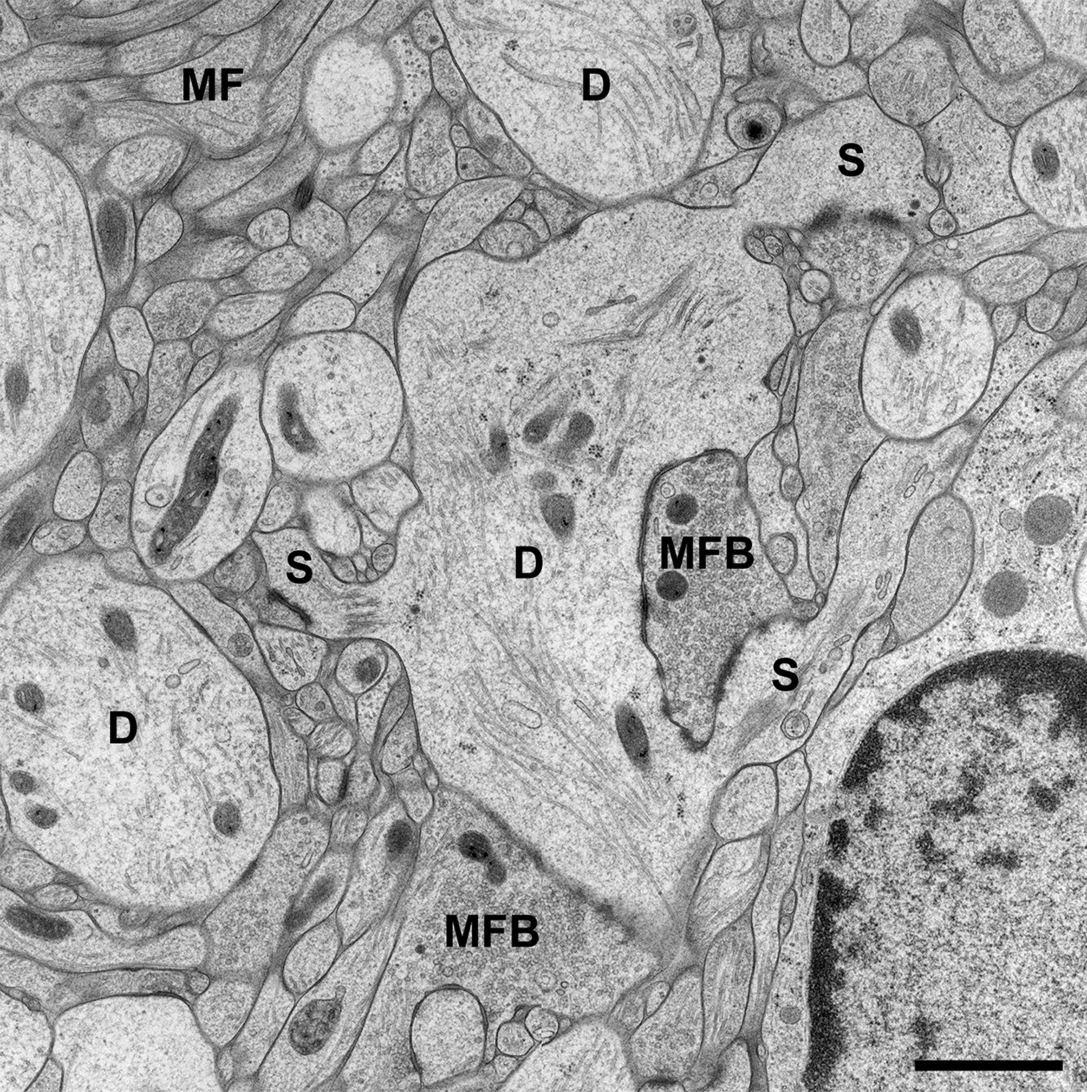
**CA3 region of Hippocampus in a slice culture incubated for 2 weeks *in vitro* (DIV).** The tissue was high-pressure frozen in the absence of chemical fixatives, subjected to freeze substitution, osmicated, and finally embedded in Epon (see Studer et al., 2014, for details on the method). Note that all tissue components of *stratum lucidum* are well preserved after the incubation period and subsequent freezing procedure. As known from many studies in perfusion-fixed material, the *stratum lucidum* mainly contains the thin unmyelinated axons of the mossy fibers (MF) and their giant boutons (mossy fiber bouton, MFB). The postsynaptic elements, the proximal dendrites (D) of CA3 pyramidal cells, are located in between the bundles of mossy fibers and give rise to large complex spines (S) for the contact with MFBs. Scale bar: 1 μm.

**FIGURE 2:**
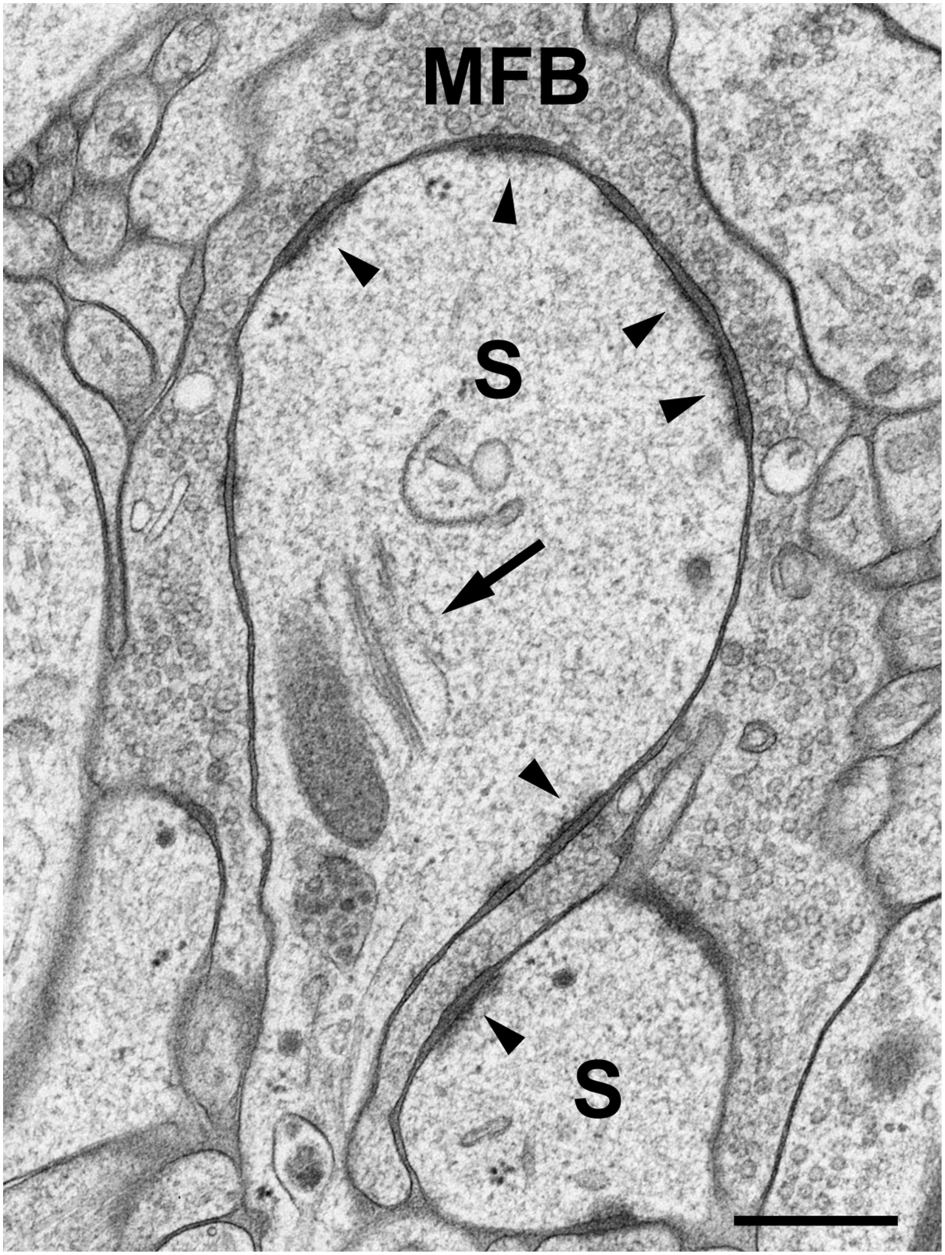
**High-pressure frozen slice culture after 2 weeks of incubation *in vitro*.** Freeze substitution, osmication, and embedding in Epon. Large complex spines (S) of a CA3 pyramidal cell dendrite, engulfed by a large MFB. The arrow points to a spine apparatus with sacs of smooth endoplasmic reticulum and electron-dense bars. Arrowheads indicate active zones. Note the widening of the extracellular space at synaptic sites and prominent postsynaptic densities that extend fine filiform protrusions into the spine cytoplasm. Occasionally, ribosomes are seen near active zones. Scale bar: 200 nm.

Brain biopsies and acute brain slices have successfully been subjected to HPF (Studer et al., 2014). Optimal results were obtained with slice cultures (Zhao et al., 2012a,b; Studer et al., 2014). In contrast to acute slices, in which numerous neurons and their processes are acutely damaged by slice preparation, the tissue is allowed to recover and reorganize in slice cultures during incubation *in vitro*, and tissue debris is removed, thus resulting in flattening of the slice. When prepared, slice cultures are about 300 μm in thickness and flatten to about 200 μm during an incubation period of 1–2 weeks. Slice cultures are thus optimal for HPF since they are sufficiently thin to be completely frozen without the formation of ice crystals. Moreover, tissue-specific characteristics are nicely preserved in this culture model (“organotypic” slice cultures), even after extended periods of incubation *in vitro*. We recognized all known tissue components and good fine-structural preservation of *stratum lucidum* of the hippocampal region CA3 in slice cultures subjected to HPF. The unmyelinated mossy fiber axons, their giant expansions, and their postsynaptic complex spines (*excrescences*) originating from proximal dendrites of CA3 pyramidal neurons, were clearly discernible under these conditions (Zhao et al., 2012a,b; Studer et al., 2014; **Figure 1**). With regard to dendritic spines, we noticed an excellent preservation of their synaptic contacts and prominent asymmetric membrane specializations, the characteristic widening of the extracellular space at active zones (synaptic cleft), and organelles such as ribosomes and the spine apparatus (**Figure 2**). Moreover, we were able to monitor subtle changes in spine fine structure associated with the induction of chemical LTP (cLTP) by using slice cultures that were immediately subjected to HPF following the experiment (Zhao et al., 2012a). Other investigators that applied HPF to the study of the nervous system observed filamentous projections from the postsynaptic membrane specialization, linking the postsynaptic density to the actin cytoskeleton (Rostaing et al., 2006), and they studied the three-dimensional architecture of the presynaptic terminal matrix (Siksou et al., 2007).

## cLTP-INDUCED FORMATION OF SMALL SPINES AT HIPPOCAMPAL MOSSY FIBER SYNAPSES

Long-term potentiation can be induced in various ways. Chemical LTP is advantageous when using HPF since the tissue can be incubated in the drug for a certain period of time and then be shock-frozen without any additional manipulation. In contrast, electrical stimulation requires the removal of the tissue from the recording chamber and transfer to the high-pressure freezer, which includes a time delay. Moreover, electrical stimulation affects an indeterminate number of synapses, whereas a large fraction of synapses are stimulated when using cLTP (Hosokawa et al., 1995). In the studies reported on here, we induced cLTP by exposing the tissue, organotypic slice cultures of hippocampus, to 25 mM tetraethylammonium (TEA) for 10 min. Control tissue was exposed to the control medium during that period of time. Whole-cell patch-clamp recordings from TEA-exposed CA3 pyramidal neurons, the target cells of hippocampal mossy fibers (MF), did indeed show strong potentiation of excitatory postsynaptic potentials (EPSPs) when compared to recording under control conditions (Zhao et al., 2012a,b). Following TEA treatment and incubation in control medium, respectively, the tissue was immediately shock-frozen using an EM PACT2 high-pressure freezer from Leica Microsystems (Vienna). The material was then subjected to cryosubstitution and embedding in Epon or Lowicryl HM20 (Zhao et al., 2012a,b; Studer et al., 2014). Epon embedding was used for studies on structural changes at spine synapses, whereas Lowicryl embedding was preferred in immunogold labeling experiments.

In our analysis, we focused on the easily identifiable mossy fiber synapse in the hippocampus formed between axons of dentate granule cells and large complex spines on proximal dendrites of CA3 pyramidal neurons (Blackstad and Kjaerheim, 1961; Hamlyn, 1962). When studying TEA-exposed tissue and control tissue in the electron microscope, several observations were made. First, we noticed small omega-shaped invaginations of the presynaptic membrane of MFBs following TEA treatment. Most likely, these membrane invaginations represent vesicle fusions with the presynaptic membrane as a result of the strong TEA-induced stimulation. In fact, quantitative assessment of the number of vesicles in stimulated and non-stimulated tissue revealed a significant decrease in the number of vesicles in MFBs of TEA-treated cultures when compared to control cultures (Zhao et al., 2012a). We interpreted this decrease in synaptic vesicles, together with an increased number of fusion events, as resulting from stimulation, and expected to see an increase in the length of the presynaptic MFB membrane. Indeed, while there was no difference in the area of MFB profiles, there was an increase in the ratio of mossy fiber perimeter/mossy fiber area, indicating a more labyrinthine course of the presynaptic membrane in stimulated mossy fiber synapses. On the postsynaptic side, we observed an increase in the complexity of the large spines or *excrescences* in contact with MFBs. In particular, we observed the formation of small, filopodia-like protrusions originating from the large complex spines (Zhao et al., 2012a,b). Thus, the more labyrinthine course of the presynaptic membrane was accompanied by a more convoluted appearance of the postsynaptic spine surface. These results confirmed and extended previous work on experience-dependent growth of mossy fiber synapses observed in light microscopic studies (Galimberti et al., 2006, 2010). The formation of these filopodia-like protrusions was accompanied by an increase in the number of synaptic contacts. Collectively, these findings indicated growth of mossy fiber synapses in response to intense stimulation. Moreover, the results showed that HPF combined with EM is a suitable method of capturing such activity-induced changes at spine synapses with high resolution. The reader is referred to our original paper (Zhao et al., 2012a) for the complete data set and to our protocol for details on the methods (Studer et al., 2014).

Appropriate controls are an important issue in studies on synaptic plasticity. As mentioned, TEA-stimulated slice cultures of hippocampus were compared to non-stimulated cultures. In addition, we used slice cultures from Munc13-1 mouse mutants that are impaired with respect to vesicle priming and docking (Augustin et al., 1999). Indeed, when we compared TEA-stimulated Munc13-1 slice cultures with non-stimulated cultures from these mutants, no differences were observed with respect to the formation of the filopodia-like spine protrusions and number of synaptic contacts (Zhao et al., 2012a). Also, there was no difference in the number of synaptic vesicles and the perimeter of the presynaptic bouton membrane between stimulated and non-stimulated cultures of Munc13-1 mutant mice.

## CHANGES IN COFILIN PHOSPHORYLATION INDUCED BY cLTP

Tetraethylammonium-induced stimulation resulted in the formation of small spines and new synaptic contacts. However, when measuring the length of these synaptic contacts, we noticed that they were shorter than in controls (Zhao et al., 2012a). We regarded this as a hint that the spine-like protrusions and their synaptic contacts were still in the process of growth after the 10-min TEA stimulation. Restructuring of spines requires remodeling of the actin cytoskeleton, which is particularly enriched in dendritic spines (Fischer et al., 1998, 2000; Matus, 2000; Star et al., 2002; Fukazawa et al., 2003; Okamoto et al., 2004; Hotulainen et al., 2009; Hotulainen and Hoogenraad, 2010). Remodeling of the actin cytoskeleton involves active, actin-depolymerizing (non-phosphorylated) cofilin to build new actin filaments and change spine shape. Accordingly, levels of p-cofilin might be decreased in early phases of LTP induction and may be associated with spine restructuring.

**FIGURE 3:**
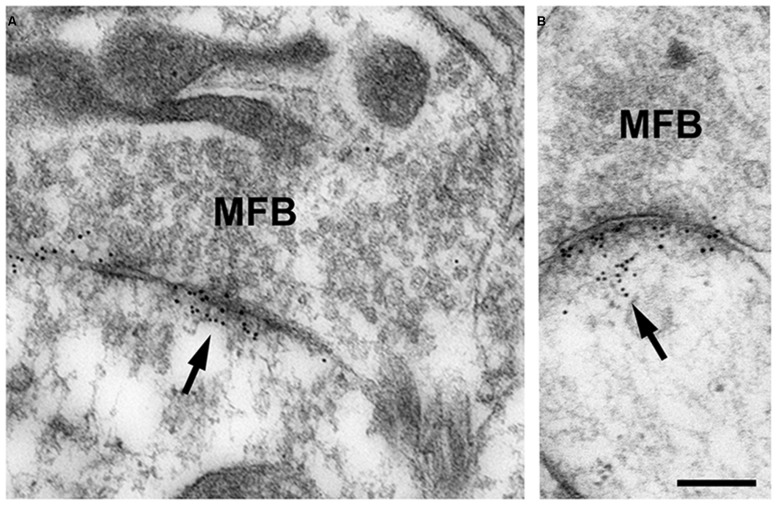
**(A,B)** High-pressure frozen slice culture of hippocampus incubated *in vitro* for 2 weeks. Freeze substitution, embedding of the tissue in Lowicryl HM20 (no osmication), and postembedding immunogold labeling for p-cofilin in thin sections of the CA3 region. Note the specific accumulations of gold grains (arrows) at synaptic sites of MFB. Only occasionally are gold grains seen at other locations. Thin sections were incubated in rabbit anti-p-cofilin (ser3; 1:100; Santa Cruz Biotechnology, Heidelberg, Germany); secondary antibodies were gold-labeled (goat-anti-rabbit, 10 nm gold grains; British Biocell, Cardiff, UK). Sections were post-stained with uranyl acetate and lead citrate. For control, the primary antibody was omitted; no specific staining was seen under these conditions. Scale bar: 200 nm.

**FIGURE 4:**
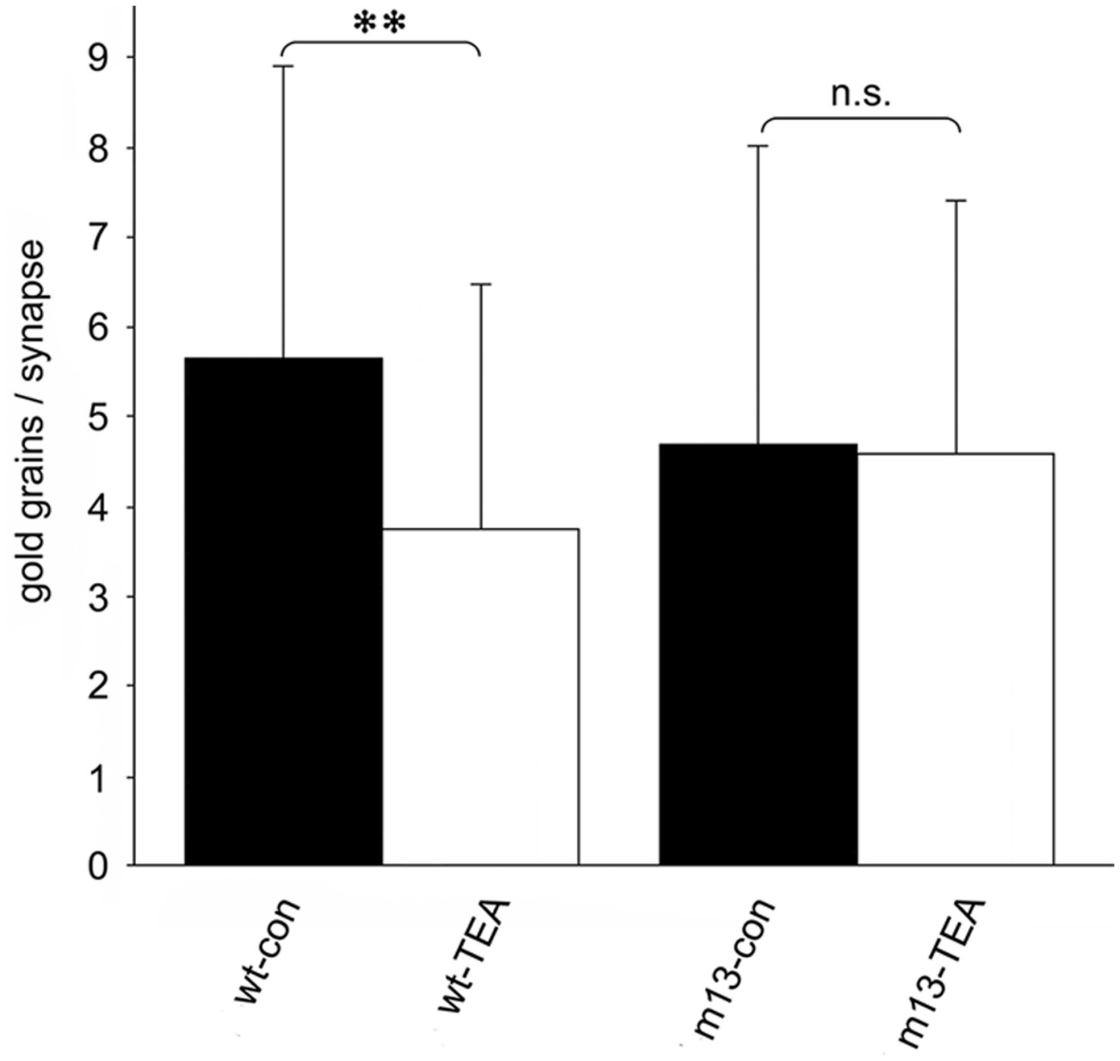
**Quantitative estimation of the number of gold grains per active zone at mossy fiber synapses in *stratum lucidum* of CA3 in hippocampal slice cultures subjected to HPF, freeze substitution, embedding in Lowicryl HM20 (no osmication), thin-sectioning, and immunogold labeling for p-cofilin.** The number of gold grains at mossy fiber synapses is significantly higher in wild-type control cultures (wt-con) compared to wt cultures treated with TEA (wt-TEA; mean ± SD; **p < 0.01). This difference between control tissue and TEA-treated tissue was not observed in cultures from Munc13-1 mutants (m13-con and m13-TEA, respectively). n.s., not significant. All gold grains within 100 nm of the postsynaptic density were regarded as specific labeling and were counted (60 active zones per experimental group; two-sided Student’s *t*-test; α = 0.05). See Studer et al. (2014) for details on the method.

In our studies on structural synaptic plasticity of mossy fiber synapses we used HPF and postembedding immunogold labeling for p-cofilin following cLTP induction by TEA. Consistent with previous EM immunogold studies for cofilin (Racz and Weinberg, 2006), we noticed accumulations of gold grains at synaptic contacts (**Figure 3**; Studer et al., 2014). Interestingly enough, we found a statistically significant decrease in the number of gold grains at mossy fiber synapses of TEA-stimulated slice cultures when compared to non-stimulated cultures (**Figure 4**). In TEA-stimulated cultures the number of gold grains at active zones of mossy fiber synapses, up to a distance of 100 nm from the membrane specialization, amounted to 3.7 ± 2.7 SD compared to 5.6 ± 3.0 SD in the control cultures. This result suggested a relative increase in non-p-cofilin and hence active cytoskeletal reorganization at the time point of immobilization by HPF. Remarkably, this decrease in p-cofilin immunoreactivity was not observed in TEA-stimulated slice cultures from Munc13-1 mutant mice (TEA: 4.5 ± 2.8 SD; control: 4.6 ± 3.3 SD; **Figure 4**), consistent with the lack of TEA-induced structural changes at spines in slice cultures from these mutants (Zhao et al., 2012a). Our results are in line with other studies pointing to an involvement of cofilin in changes in spine structure and reorganization of the actin cytoskeleton associated with LTP (Fukazawa et al., 2003; Lisman, 2003; Zhou et al., 2004; Chen et al., 2007; Rex et al., 2009). In mutants deficient in LIM kinase-1 (LIMK-1), the kinase that phosphorylates cofilin, LTP is enhanced associated with changes in spine morphology and a reduced length of the postsynaptic density (Meng et al., 2002).

An interesting side effect of the immunogold labeling studies was that, in general, the number of gold grains indicating p-cofilin immunoreactivity was much larger in slice cultures subjected to HPF, compared to cultures conventionally fixed using aldehyde solutions. It is of note that this was not due to increased background staining (Studer et al., 2014). These observations point to an increased signal-to-noise ratio of immunolabeling following freezing, freeze substitution, and embedding in Lowicryl, as similarly described in previous studies (e.g., Moreira et al., 1998). This is likely because protein denaturation by aldehydes and robust dehydration in ethanol are avoided. Thus, HPF combined with freeze substitution and Lowicryl embedding may be a useful alternative to conventional EM immunocytochemical methods.

## MOSSY FIBER BOUTONS AND THEIR POSTSYNAPTIC SPINES ARE RESTRUCTURED IN AN ACTIVITY-DEPENDENT MANNER

Numerous studies, including those on the famous patient H.M., have indicated that the hippocampal formation plays an important role in learning and memory processes. What does this mean at the level of cells, projections, and synapses? Sensory perception of novel, probably dangerous changes in the environment will require behavioral adjustment to cope with the new situation. In higher centers, such as the cerebral cortex and hippocampus, behavioral adjustment involves associations and recall of previous, similar situations. It is generally assumed that this “learning” about a novel environment is achieved by modification of the neuronal circuit. Information about the environment is fed into the hippocampus via the entorhinal cortex, which receives input from a large variety of sensory centers. Circuit modification is then assumed to take place in the hippocampus, particularly in the synapses of the trisynaptic pathway (Andersen et al., 1971), involving the synapses of entorhinal fibers with dentate granule cells, granule cell synapses (mossy fiber synapses) with CA3 pyramidal neurons and mossy cells, and CA3 pyramidal cell projections (Schaffer collaterals) to CA1 pyramidal neurons. Plasticity of transmission at hippocampal synapses, including changes in the structure of spines and synaptic contacts as seen in LTP or LTD (long-term depression), has accordingly become a widely studied cellular model of learning and memory processes.

Potentiation or depression of synaptic transmission is associated with molecular and structural changes at synapses. In this review, we report on the formation of finger-shaped protrusions emerging from the complex spines of mossy fiber synapses and *de novo* formation of active zones in response to cLTP induction. These structural changes were associated with decreased phosphorylation of cofilin, suggesting active remodeling of the actin cytoskeleton in the complex spines. It is most likely that these fine-structural and molecular changes underlie the experience-dependent increase in complexity of mossy fiber synapses observed in light microscopic studies (Galimberti et al., 2006, 2010).

## Conflict of Interest Statement

Michael Frotscher, Werner Graber, Xuejun Chai, Sigrun Nestel and Shanting Zhao declare that they have no competing financial interests. Daniel Studer was involved in the development of EM PACT and receives royalties from Leica Microsystems (Vienna, Austria).
